# Gender Associated with the Intention to Choose a Medical Specialty in Medical Students: A Cross-Sectional Study in 11 Countries in Latin America

**DOI:** 10.1371/journal.pone.0161000

**Published:** 2016-08-12

**Authors:** Luis Fernando Ng-Sueng, Iván Vargas-Matos, Percy Mayta-Tristán, Reneé Pereyra-Elías, Juan José Montenegro-Idrogo, Fiorella Inga-Berrospi, Felix Ancalli, Francisco Bonilla-Escobar, Cristian Diaz-Velez, Erick Gutierrez-Quezada, Jennifer Gomez-Alhach, Carlos E. Muñoz-Medina, Adriana Sanchez-Pozo, Milisen Vidal, Red LIRHUS

**Affiliations:** 1 Escuela de Medicina, Universidad Peruana de Ciencias Aplicadas, Lima, Perú; 2 Sociedad Científica Estudiantes de Medicina UPC, Lima, Perú; 3 Universidad Nacional Mayor de San Marcos, Lima, Perú; 4 Universidad Nacional Jorge Basadre Grohmann, Tacna, Perú; 5 Instituto Cisalva, Escuela de Salud Pública, Universidad del Valle, Cali, Colombia; 6 Universidad de San Martín de Porres, Chiclayo, Perú; 7 Universidad Autónoma de Nayarit, Tepic, Nayarit, México; 8 Universidad de San Martín de Cali, Cali, Colombia; 9 Universidad del Oriente Núcleo Bolivar, Ciudad Bolivar, Venezuela; 10 Universidad Nuestra Señora de La Paz, La Paz, Bolivia; 11 Universidad de Concepción, Concepción, Chile; National Institute of Health, ITALY

## Abstract

**Introduction:**

The selection of a medical specialty has been associated with multiple factors, such as personal preferences, academic exposure, motivational factors and sociodemographic factors, such as gender. The number of women in the medical field has increased in recent years. In Latin America, we have not found any studies that explore this relationship.

**Objective:**

To determine whether there is an association between gender and the intention to choose a medical specialty in medical students from 11 countries in Latin America.

**Methods:**

Secondary analysis of the Collaborative Working Group for the Research of Human Resources for Health (Red-LIRHUS) data; a multi-country project of students in their first year and fifth year of study, from 63 medical schools in 11 Latin American countries. All students who referred intention to choose a certain medical specialty were considered as participants.

**Results:**

Of the 11073 surveyed students, 9235 indicated the name of a specific specialty. The specialties chosen most often in the fifth year were General Surgery (13.0%), Pediatrics (11.0%), Internal Medicine (10.3%) and Obstetrics/Gynecology (9.0%). For women, the top choices were Pediatrics (15.8%), Obstetrics/Gynecology (11.0%), Cardiology (8.7%), General Surgery (8.6%), and Oncology (6.4%). In the adjusted analysis, the female gender was associated with the choice of Obstetrics/Gynecology (RP: 2.75; IC95%: 2.24–3.39); Pediatric Surgery (RP: 2.19; IC95%: 1.19–4.00), Dermatology (RP: 1.91; IC95%:1.24–2.93), Pediatrics (RP: 1.83; IC95%: 1.56–2.17), and Oncology (RP: 1.37; IC95%: 1.10–1.71).

**Conclusions:**

There is an association between the female gender and the intention to choose Obstetrics/Gynecology, Pediatrics, Pediatric Surgery, Dermatology, and Oncology. We recommend conducting studies that consider other factors that can influence the choice of a medical specialty.

## Introduction

After finishing their studies, medical students face the decision of whether to choose a medical specialty. If they choose to do so, they find a large number of options from which to choose. As some recent studies show, this choice has been associated with multiple factors, such as personal preferences[1,2], the academic exposure that the student has had[3], motivational factors[4,5], the specialty’s working conditions[6], and also some sociodemographic factors[7–9]. For this reason, the tendencies of medical specialty choice vary on the global level [6,10,11]. Similarly, many studies reveal a predominance of non-surgical specialties [1,11], whereas others show that surgical specialties are the most preferred[10,12,13]. The majority of these studies are from the United States, Europe, or Asia. However, to this date, we have not been able to find studies on this topic in Latin America.

One of the most studied sociodemographic factors with respect to the choice of medical specialty is gender, principally the female gender. There are studies that describe the current increase in the number of women who enroll in medical schools[14,15] and others that report a greater number of women graduating as doctors[16]. This recently reported fact is called the feminization of medicine[17], which denotes an interesting sociocultural change, given that before the beginning of the twentieth century, the role of women in the field of health was mainly supportive (medical assistants, nurses, etc.) [18].

Although the feminization phenomenon mentioned earlier has been studied with more emphasis in the last ten to fifteen years, the Pan American Health Organization (PAHO) explored the general characteristic of medical school students in Latin America in the sixties[19]. At that time, it was described a growing number of female medical students (21% regsistered females), however there was great difference in-between countries of the region, Venezuela had the largest number of female medical students (nearly 30 percent) and Guatemala the less (no more than 10 percent). There were even some concerns from medical educators about the growing number of females in medicine and there were suggestions to limit their acceptance into medical schools based on unfounded reasons, according to this PAHO report[19].

Currently some data of the region support this outgrowing number of female in medicine. In 1980 in Mexico, there were 33% female medical students registered in medical schools, by 1999 there were 52% and it is estimated that by 2027, this number may reach 60% [20]. Comparably, in Ecuador non-private medical schools admitted a cohort with a greater number of accepted female students (5853 vs 4276) in 2008 and, also a greater number of female graduated by 2013 [21]. In Colombia, Ruiz et al. described more female graduates in Health Sciences careers and a growing number of female graduates in medical programs (1980 to 2011) [22]. As shown, the gender gap in medical education is apparently narrowing. Furthermore, in Spain, in the 1994–2011 term, there has been a growth of 93.4% of collegiate female physicians in contrast to 12.6% of male physicians [23]. This is not a minor detail if we consider that most of the foreign physicians in Spain come from Latin America and the Caribbean [24].

Nowadays, a trend towards a preference for the main four medical specialties has been shown in European, Asian, and North American studies: Internal Medicine, General Surgery, Obstetrics/Gynecology, and Pediatrics [11,13,25]. Despite not always presenting in the same order, these specialties consistently predominate over others. Findings suggest an association between these specialties and gender [8,26,27]; however, this issue continues to be studied. With the aim of contributing with more data on this topic, the objective of our study is to evaluate the influence of gender on the choice of a medical specialty in Latin American medical students. In addition, we aim to explore the phenomenon of feminization in this population.

## Methods

### Original study

Data was collected by the Latin American Collaborative Working Group for the Research of Human Resources for Health (Red-LIRHUS), a cross-sectional multi-country study that aimed to describe the academic, motivational and professional profiles of students from first year and fifth year of medical school. It was performed in 63 medical schools in 11 Latin American countries (Argentina, Bolivia, Colombia, Chile, Ecuador, Honduras, Mexico, Panama, Paraguay, Peru, and Venezuela) [28]. The study was conducted using a self-administered questionnaire at the different participating universities from September 2011 to July 2012.

### Study design and participants

The current study is a secondary analysis of the data from the Red-LIRHUS project, and its main objective is to determine the association between gender and the intention of Latin American medical students to choose a medical specialty. The inclusion criteria were all surveyed students who declared they wish to pursue a specialty and those who referred a specific specialty. We excluded those participants who did not declare their gender in the survey.

### Outcome and independent variables

The dependent variable was the intention to choose a medical specialty. This variable was evaluated using a self-administered questionnaire with the following two questions: “Ten years after having finished medical school, do you plan to have completed a medical specialty (residency)?” and “Which specialty do you plan to develop?” [13,29] The specialties were described by frequency according to gender and year of study. The final analysis considered the most chosen specialties, with the aim of omitting those with non-significant percentages. All of the specialties were considered individually; they were not grouped (e.g., surgical specialties, clinical, etc.).

The covariables included were sociodemographic variables (age, gender, having children, and having current paid work), university-related variables (year of study, country, funding of university, and the location of the university in the capital), and variables related to the medical degree and professional trajectory (the presence of a family member who is a doctor, the expectation of the number of jobs and income in 10 years); all of these were collected in the same questionnaire.

### Statistical analysis

The database was entered into Microsoft Excel^®^ and then reviewed for quality control, refining poorly completed questionnaires and erroneous data transcription. The analysis was performed using the statistical package STATA 11.0. First, we performed a descriptive analysis of the characteristics of the population. We used the Shapiro-Wilk test to verify the normal distribution of the age; then, means and standard deviations were described. The expectation of income 10 years after graduation was categorized into tertiles. To evaluate if there were significant differences according to sex, we used the Student’s t-test for age and the chi^2^ test for categorical variables. To corroborate if there was a significant change between the intention to choose a certain specialty between students in their first year and fifth year, we used the chi^2^ test.

We calculated the crude and adjusted prevalence ratios (PR and aPR) between gender and the intention to choose a specialty using simple and multiple Poisson regression models with robust variance, respectively. The adjusted models included all of variables described above, given that these were associated with the intention to choose some of the principal specialties in previous studies [4,9,12]. The final results were given as aPR with their respective confidence intervals at a 95% level. We considered a P-value<0.01 as statistically significant.

### Ethical aspects

Ethical approval for this study was obtained from the Institutional Review Board of the Universidad Peruana de Ciencias Aplicadas. The original study was approved by the Ethics Committee of the Instituto Nacional de Salud del Perú. In each one of the schools included, the corresponding authorities approved the execution of the project; similarly, all of the participants gave their verbal informed consent.

## Results

### General Characteristics of the Population

**Fig 1:**
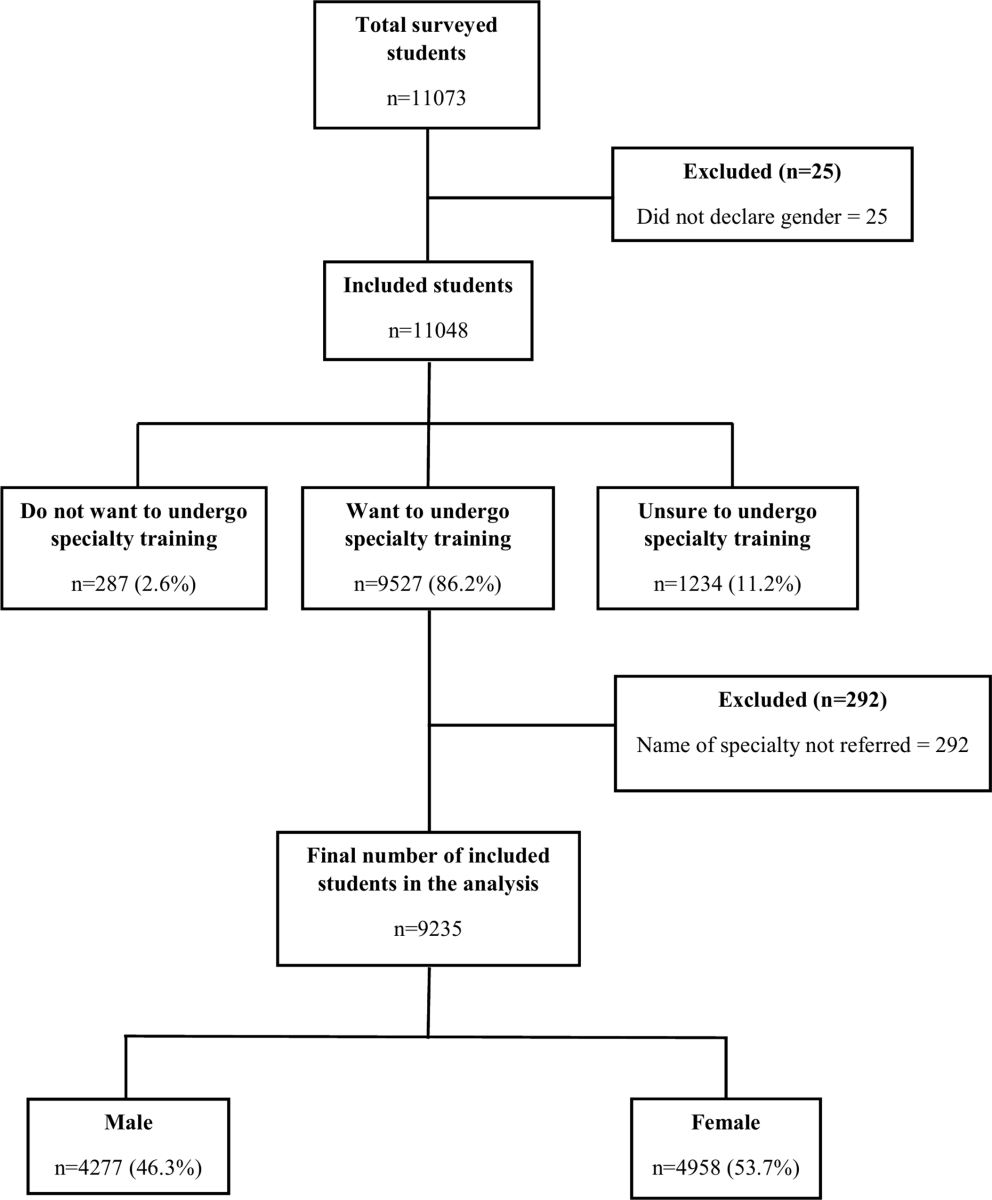
Flowchart of participants included in the study: Gender and the intention to choose a specialty in medical students from 11 countries of Latin America.

From a total of 11,073 students, 25 who did not specify their gender were excluded. The participants who did not want to pursue specialty training (n = 287), those who were unsure whether to study a specialty (n = 1,234), and those who did not indicate the name of the specialty (n = 292) were not included in the analysis (**Fig 1**). We found a significant difference between the 1,837 excluded subjects and the study population in terms of gender, having children, having a current paid work, the funding and place of the university, the year of study, the expected number of jobs, income expectations, and having family members who are doctors. About 53.7% of the 9,235 included in the study were women, with a mean age of 20.4 years. Most of them were enrolled in their first year of study (64.3%), principally from universities located outside the capital (67.0%), from publicly-funded universities (63.4%), and had family members who were doctors (51.3%), with Peru as the country with the largest proportion of participants (35.2%) **(Table 1**).

**Table 1:** Sociodemographic characteristics according to gender in medical students of 11 countries of Latin America.

Variable	Total		Male		Female		Male/ Female	p
	n	(%)	n	(%)	n	(%)		
	**9235**	**(100)**	**4277**	**(46.3)**	**4958**	**(53.7)**		
**Sociodemographic**								
Age*	20.4	(2.9)	20.7	(3.1)	20.1	(2.7)		<0.001
Having children								0.013
Yes	378	(4.1)	199	(4.7)	179	(3.7)		
No	8772	(95.9)	4045	(95.3)	4727	(96.4)		
Year of study								0.016
First	5939	(64.3)	2695	(63.0)	3244	(65.4)	1.20	
Fifth	3296	(35.7)	1582	(37.0)	1714	(34.6)	1.08	
Current paid work ¶								<0.001
Yes	800	(8.7)	497	(11.7)	303	(6.2)	0.60	
No	8387	(91.3)	3760	(88.3)	4627	(93.8)	1.23	
**University**								
Country								<0.001
Bolivia	1429	(15.5)	607	(42.5)	822	(57.5)	1.35	
Chile	487	(5.3)	257	(52.8)	230	(47.2)	0.89	
Colombia	1291	(13.9)	601	(46.6)	690	(53.5)	1.14	
Costa Rica	138	(1.5)	60	(43.5)	78	(56.5)	1.30	
Ecuador	517	(5.6)	290	(56.1)	227	(43.1)	0.78	
El Salvador	89	(1.0)	40	(44.9)	49	(55.1)	1.22	
Honduras	924	(10.0)	412	(44.6)	512	(55.4)	1.24	
México	188	(2.0)	99	(52.7)	89	(47.3)	0.89	
Paraguay	127	(1.4)	61	(48.0)	66	(52.0)	1.08	
Perú	3253	(35.2)	1578	(48.0)	1675	(52.0)	1.06	
Venezuela	792	(8.6)	272	(34.3)	520	(65.7)	1.91	
Located in the capital								<0.001
Yes	3047	(33.0)	1296	(30.3)	1751	(35.3)	1.35	
No	6188	(67.0)	2981	(69.7)	3207	(64.7)	1.07	
Private								<0.001
Yes	3379	(36.6)	1483	(34.7)	1896	(38.2)	1.27	
No	5856	(63.4)	2794	(65.3)	3062	(61.8)	1.12	
**Career**								
Family member who is a doctor ¶								0.206
Yes	4722	(51.3)	2155	(50.6)	2567	(59.9)	1.19	
No	4479	(48.7)	2103	(49.4)	2376	(48.1)	1.12	
# of jobs in 10 years								<0.001
One	1482	(16.6)	730	(17.7)	752	(15.7)	1.03	
Two	5570	(62.4)	2429	(58.8)	3141	(65.4)	1.29	
Three	1226	(13.7)	587	(14.2)	639	(13.3)	1.08	
More than three	652	(7.30)	383	(9.30)	269	(5.6)	0.45	
Expectation of income in 10 years¶								<0.001
< = 2000 $	2524	(38.2)	1170	(34.8)	1354	(41.8)	1.15	
2001–5000 $	2434	(36.9)	1255	(37.4)	1179	(36.4)	0.93	
> 5000 $	1641	(24.9)	933	(27.8)	708	(21.8)	0.75	

*Mean and standard deviation

¶ Present some missing data

### Specialty choice intentions

The medical specialties with the highest choice intentions in first year were Pediatrics (13.3%), Cardiology (12.8%), General Surgery (7.9%) and Neurology (7.8%). The preferences of subjects in their fifth year were General Surgery (13.0%) most often, followed by Pediatrics (11.0), Internal Medicine (10.3%), and Obstetrics and Gynecology (9.0%) **(Table 2)**.

**Fig 2:**
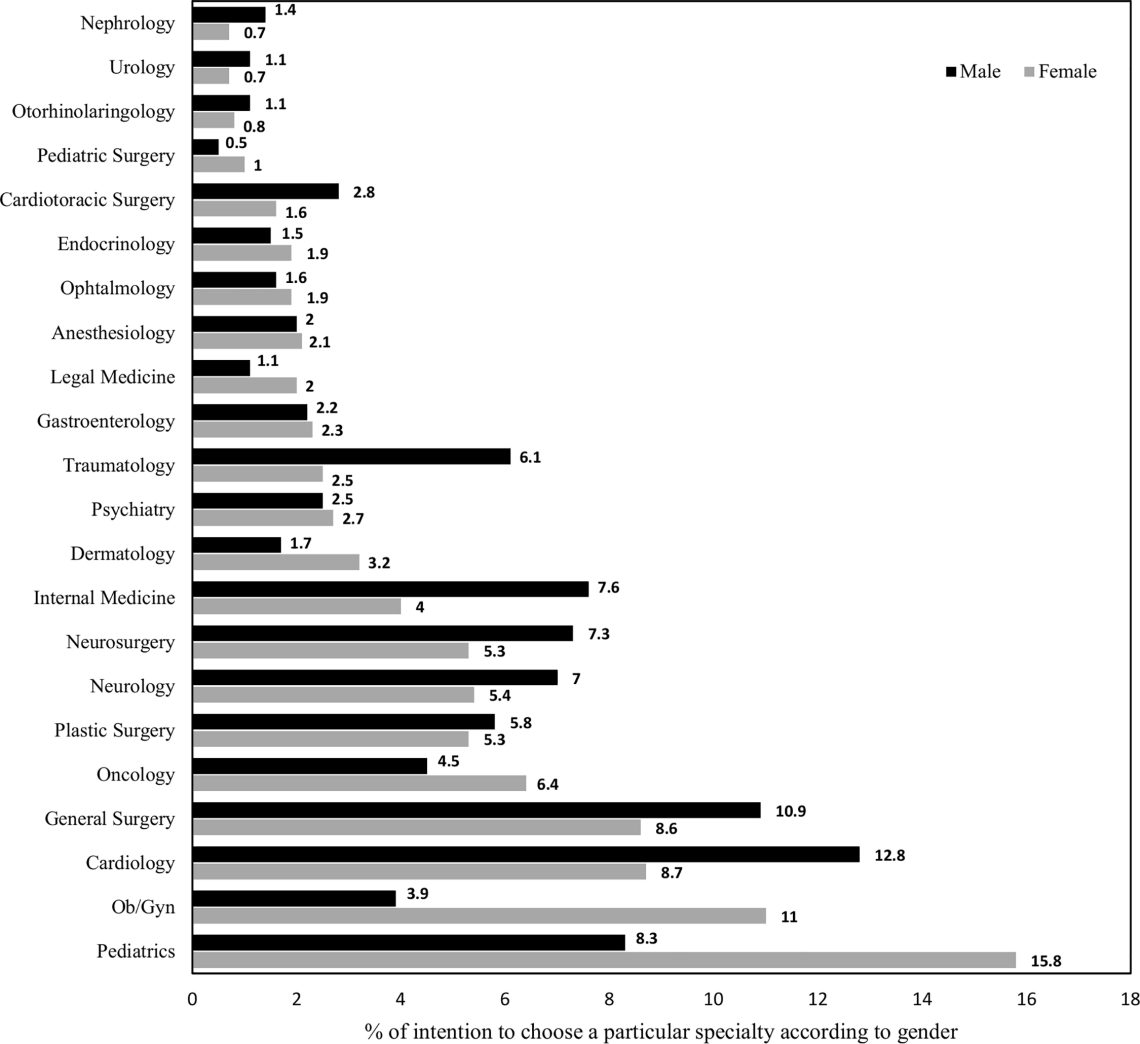
Frequency of intention to choose a particular specialty according to gender in medical students of 11 countries of Latin America.

For women, the five most frequently chosen specialties were Pediatrics (15.8%), Obstetrics and Gynecology (11.0%), Cardiology (8.7%), General Surgery (8.6%), and Oncology (6.4%). These coincided with three of the five specialties most frequently chosen by the male gender (Pediatrics, Cardiology, and General Surgery) **(Fig 2)**.

**Table 2:** Proportions of intention to choose a particular medical specialty according to year of study and its direction of change in medical students of 11 countries of Latin America.

#	First year	(%)	#	Fifth year	(%)	Δ
1	Pediatrics	13.3	1	General Surgery	13.0	↑
2	Cardiology	12.8	2	Pediatrics	11.0	↓
3	General Surgery	7.9	3	Internal Medicine	10.3	↑
4	Neurology	7.8	4	Obstetrics & Gynecology	9.0	↑
5	Neurosurgery	7.7	5	Cardiology	6.7	↓
6	Obstetrics & Gynecology	7.0	6	Traumatology	4.4	↑
7	Oncology	6.8	7	Plastic Surgery	4.0	↓
8	Plastic Surgery	6.4	8	Neurosurgery	3.6	↓
9	Traumatology	4.0	9	Dermatology	3.4	↑
10	Internal Medicine	3.1	10	Neurology	3.2	↓
11	Psychiatry	2.6	11	Oncology	3.1	↓
12	Legal Medicine	2.0	12	Gastroenterology	2.8	↑
13	Dermatology	2.0	13	Anesthesiology	2.6	↑
14	Cardiothoracic Surgery	2.0	14	Psychiatry	2.4	↓
15	Gastroenterology	1.9	15	Cardiothoracic Surgery	2.3	↑
16	Ophthalmology	1.6	16	Endocrinology	2.3	↑
17	Anesthesiology	1.6	17	Ophthalmology	2.0	↑
18	Endocrinology	1.4	18	Nephrology	1.9	↑
19	Otorhinolaryngology	0.7	19	Urology	1.5	↑
20	Urology	0.6	20	Otorhinolaryngology	1.5	↑
21	Nephrology	0.6	21	Pediatric Surgery	1.1	↑
22	Pediatric Surgery	0.6	22	Infectious diseases	1.1	↑
23	Infectious diseases	0.3	23	Legal Medicine	0.8	↓

Δ Direction of change according to year of study (first to fifth year).

### Association between gender and chosen specialty

In the crude analysis, the female gender was associated with the choice of Obstetrics/Gynecology, Pediatrics, Pediatric Surgery, Dermatology, and Oncology. In the adjusted analysis, the female gender presented the strongest association with the choice of Obstetrics/Gynecology (p<0.001; RP: 2.75; IC95%: 2.25–3.39). Similarly, the association women were more likely to refer the intention to pursue specialized training in Pediatric Surgery (p = 0.011; RP: 2.19; IC95%: 1.19–4.00), Dermatology (p = 0.005; RP:1.90; IC95%: 1.24–2.93), Pediatrics (p<0.001; RP: 1.83; IC95%: 1.56–2.17), and Oncology (p = 0.005; RP: 1.37; IC95%: 1.09–1.71). At the other pole, female participants were less likely to choose General Surgery, Neurology, Neurosurgery, Cardiology, Internal Medicine, Thoracic Surgery, Traumatology/Orthopedics and Infectious Diseases **(Table 3)**.

**Table 3:** Association between female gender and intention to choose a particular medical specialty in medical students of 11 countries of Latin America.

Specialty	Crude analysis †			Adjusted analysis *†		
	PR	(95%CI)	p	PR	(95%CI)	p
Obstetrics & Gynecology	2.83	(2.39–3.35)	<0.001	2.75	(2.25–3.39)	<0.001
Pediatric Surgery	2.00	(1.22–3.29)	0.006	2.19	(1.19–4.00)	0.011
Dermatology	1.92	(1.46–2.52)	<0.001	1.91	(1.24–2.93)	0.005
Pediatrics	1.91	(1.70–2.15)	<0.001	1.83	(1.56–2.17)	<0.001
Oncology	1.44	(1.21–1.71)	<0.001	1.37	(1.10–1.71)	0.005
Endocrinology	1.23	(0.90–1.69)	0.189	1.22	(0.83–1.79)	0.307
Anesthesiology	1.07	(0.83–1.38)	0.737	1.16	(0.79–1.73)	0.448
Ophthalmology	1.21	(0.89–1.65)	0.229	1.16	(0.84–1.60)	0.355
Gastroenterology	1.04	(0.79–1.36)	0.792	1.09	(0.76–1.58)	0.650
Psychiatry	1.07	(0.83–1.38)	0.578	0.96	(0.76–1.21)	0.748
Plastic Surgery	0.91	(0.77–1.08)	0.298	0.92	(0.74–1.16)	0.513
Otorhinolaryngology	0.70	(0.46–1.07)	0.098	0.78	(0.43–1.39)	0.395
Urology	0.64	(0.41–1.00)	0.047	0.83	(0.55–1.26)	0.384
Nephrology	0.51	(0.34–0.77)	0.001	0.66	(0.41–1.06)	0.086
General Surgery	0.78	(0.69–0.89)	<0.001	0.82	(0.71–0.96)	0.014
Neurology	0.77	(0.65–0.90)	0.001	0.75	(0.61–0.92)	0.005
Neurosurgery	0.71	(0.61–0.84)	<0.001	0.72	(0.59–0.89)	0.002
Cardiology	0.68	(0.60–0.76)	<0.001	0.70	(0.60–0.83)	<0.001
Internal Medicine	0.52	(0.44–0.63)	<0.001	0.54	(0.45–0.67)	<0.001
Cardiothoracic Surgery	0.56	(0.42–0.74)	<0.001	0.48	(0.34–0.67)	<0.001
Traumatology	0.41	(0.34–0.50)	<0.001	0.44	(0.33–0.59)	<0.001
Infectious diseases	0.44	(0.26–0.77)	0.004	0.33	(0.18–0.60)	<0.001

*Adjusted by age, having children, year of study, having current paid work, country, type of university, location of university in the capital, family member who is a doctor, income 10 years after graduation; cluster analysis by university.

† n = 9235

## Discussion

### Student profile and trend towards feminization

**Fig 3:**
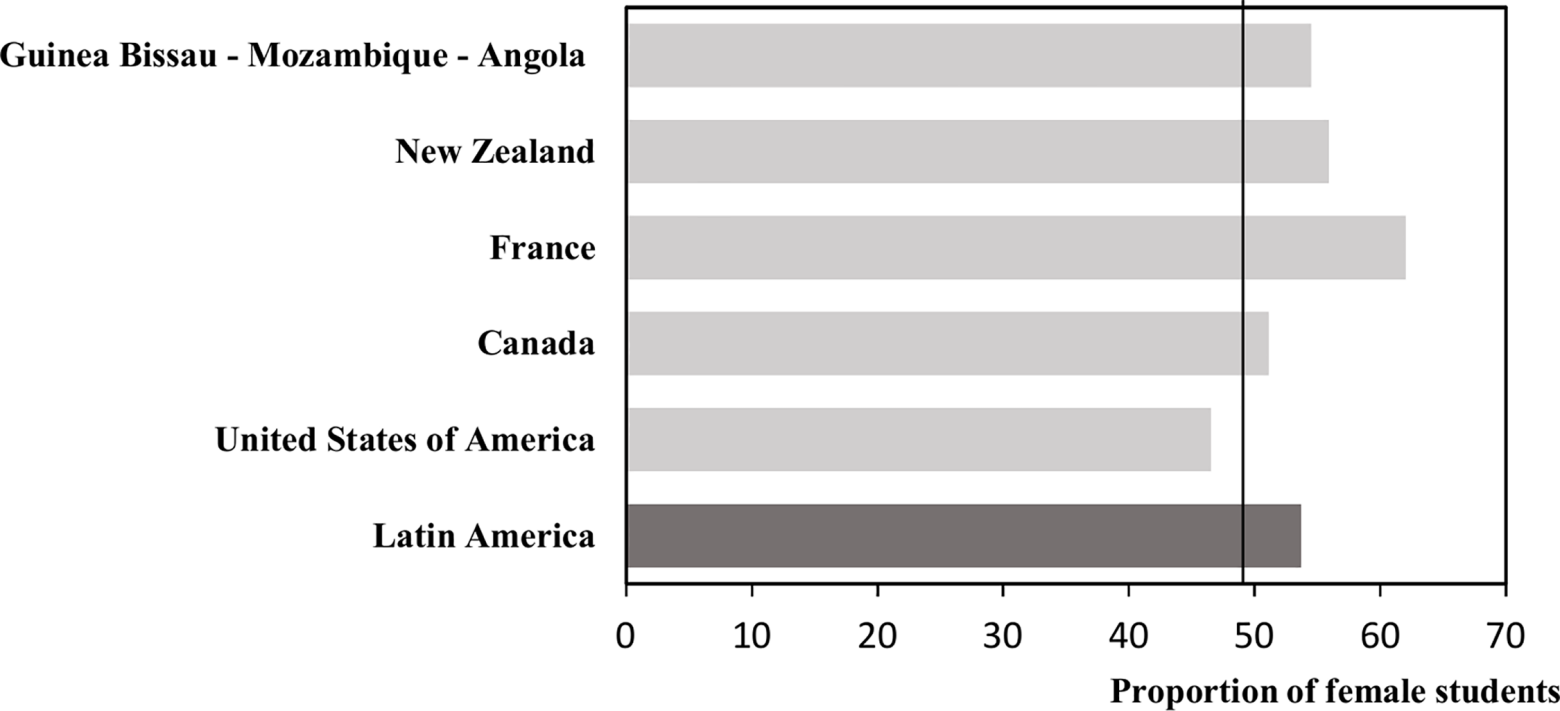
Proportion of female medical students in national or multi-country studies [14,16,24,33,35].

One of the most important aspects of this current profile of Latin American medical students is the number of women enrolling in medical schools. This aspect correlates with what has been described in countries such as the United States, Canada and New Zealand [14,16,30] **(Fig 3)**. In the present study, only three of the eleven countries analyzed had a higher proportion of male students, which represents the phenomenon of feminization. In other studies at Europe[17], Asia[31], and Latin America[32,33], a growing number of women in medical schools has been reported. In the year 2003, the proportion of female doctors in Peru was about 32% of all graduates and 45.5% by the year 2007. The present study shows the current proportion of Peruvian and Latin American women medical students corresponding to 52% and 53.7% of all students respectively[33]. Possible explanations for this apparently global phenomenon are the increasing importance that women have gained in Latin American society, access to higher education[32] (professional degrees, master’s degrees and doctoral training) [34] and to the improvement in job opportunities[35].

Most women enrolling in medical schools are from private universities located in the capital, which may denote better economic access, a factor that would explain the equality of opportunities noted above. However, if there is already equality in the conditions to develop a professional career such as medicine, it is necessary to explain why the proportion of women in this field is still growing. Studies have reported that if women’s interest in medicine has increased, men have apparently lowered their levels of preference or have remained unchanged. Possibly, men may prefer fields related to business [36] and information technology development[37].

### Intention to choose a medical specialty

The ranking of specialties of students in their fifth year correlates with multiple studies around the world[12,25,38,39]. Although they do not coincide in strict order of preference with our findings, the five most chosen specialties are repeated globally. General Surgery was the preferred specialty in our study, which is the same as the findings in two European studies and one Australian study[39–41]. However, Internal Medicine and Pediatrics were the most chosen in studies in other continents[27,38,42]. Finally, Obstetrics/Gynecology and Cardiology were also among the most chosen in other studies; although they may not be the first choice, these tend to appear among the most preferred[38,40]. Apparently, there is a preference for general specialties but not subspecialties.

Nevertheless, it is necessary to emphasize the difference in choice found between students in their first year and fifth year. It is evident that preferences are dynamic according to the year of study[43],which can be explained by different factors. One factor is related to the experiences of the students as they are exposed to the full spectrum of different specialties in the medical curricula [43,44]. Other reason for this variation have been attributed to the model that different professors represent to students [45,46]. Similarly, Girasek et al. found that first-year students consider the choice of specialty in accordance to the economic income it offers [47].

However, the differences found in the specialty ranking between the fifth-year students in our study, compared to others, could be due to multiple causes. Two studies have concluded that the trends in the choices of medical students in developing countries could be conditioned by the search for better living conditions, such as in the case of some Latin American countries[48,49]. These results could explain the tendency of students in developed countries to undergo specialties considered less well-paying[6,50] because they may not face the probable economic necessity of students in low-income countries. Are et al. found that students from low-resource settings expressed a greater interest in surgical fields compared to those from developed countries[26]. Similarly, Gibis et al. identified an association between the choice of specialty and the salary expectation of the student[6], whereas a Spanish study found another strong association with the availability of job positions in determining preferences[51].

### Gender

The finding that Obstetrics and Gynecology is the specialty with the greatest association with the female sex is congruent with various different studies[4,13,29]. The explanation for this finding could be related to those from Schnuth et al., who found an association between a woman’s health, the possibility of improving it, and the choice of this specialty[52]. In the same way, it is possible that the cause also resides in women’s identification with maternity. In their study, Wendell et al. found that the most popular specialty for women with children is Obstetrics/Gynecology[53]. The fact that there is an association between the female gender and this specialty could explain the growing number of female obstetricians/gynecologists that is being reported[4]. The presence of mentors who are mostly women[4,54] could be generating a feedback loop that increases the number of women in this specialty.

The other specialties found to be associated with the female gender are Pediatric Surgery, Pediatrics, Oncology, and Dermatology. It is possible that this association could be explained by the inherent characteristics that a woman brings to the medical career, such as compassion, empathy, warm communication, and amicability[55,56]. Nevertheless, another factor to take into account is the balance a woman would have to make between the medical career and her family (maternity leave, time for breastfeeding, childbearing, etc.). Burton et al. noted that in Canada, women work fewer hours, see a lower amount of patients, have a higher probability of leaving medicine sooner, and join a greater proportion of professional organizations in comparison to men[16]. These aspects are correlated with specialties such as Dermatology and Oncology, which do not frequently have emergency scenarios or long hospital rounds but instead more hours in offices and consultations, offering more lifestyle flexibility.

With respect to the male gender, we found an association mainly with General Surgery and other surgical specialties, which is consistent with multiple studies[4,9,29,40,57,58]_._ The surgical sciences continue to be dominated by men[9,57], most likely because of the social stereotype that exists in the medical environment, which promotes a type of “male surgeons’ club” [59]_._ Surgery has been related to prestige, social status, and better professional opportunities[29]. In our results, men have higher economic expectations and desire to have a greater number of jobs. It is interesting that General Surgery is considered to belong to the male gender not only by men but also by women, who do not prefer it because of these potentially segregating stereotypes[60] or because of prior negative experiences, such as discriminatory attitudes when performing training rotations in General Surgery[59]. However, the lack of scheduling flexibility and the difficulty in maintaining a family balance could also distance women from this specialty.

Obstetrics/Gynecolgy and Pediatric Surgery are surgical specialties; however there is no negative stereotype that distances women. Similarly, it would seem that the characteristics of identification with maternal-infant health and the more amicable and humanitarian character prevail over how demanding this fields could be[59]. Nevertheless, the authors believe that these factors do not fully explain this phenomenon; thus, further study is necessary.

#### Women in medicine: Why does it matter?

There are several studies addressing the possible outcomes of the feminization phenomenon in medicine[17,19,36,61,62]. This has led to direct research studies to define women’s profile and position in medicine, as in this study. First of all, it is important to objectify the global growth of women in medicine because there still gender gap in the profession. There is data that shows women earn lower salaries than men, even in high positions in medicine [63–65]and are less represented in high positions as well[66,67]. This might not be a minor detail if we consider that specialties with high number of female physicians (or high intention to be chosen) like Obstetrics and Gynecology as stated in this paper, are not being represented as expected [66].

This relatively “new” panorama brings some social issues to the table about the female and male role in society. As stated by Boulis A and Jacobs J, women have entered a profession designed for the “male bread-winner and the stay-at-home spouse” [36]. This might be reflected in some of our results in which Latin American female medical students expect to earn less and have less jobs in 10 years than male students, although the gap is narrow. Even more, in a doctoral thesis in Ecuador, medical graduates and medical specialists were interviewed and it was found that some male physicians believed that child raising was almost exclusively a woman’s task and female physicians that were mothers at the time, believed that child raising was more important as their medical career[21]. Some authors suggest that, even though maternity permission is to be granted for female physicians, some mechanism must be created to apply it for both sexes to promote equality and not to “penalize” only women in this career [61].

Also, there is a concern about balancing the workforce [62]. As previously discussed, women are more likely to have longer periods of absent during their career development due to pregnancy and maternity [16]. As reinforced by this paper, there are gender-based choices in certain specialties, which eventually could lead a misbalance in human health resources. This must be a concern, especially in Latin America where a lack of medical specialists has been described in some countries like Peru [68]. There is an urge to change policies and strengthen medical residents training, in order to attract more medical trainees regardless of their gender, so they can satisfy the demand [69]. Thus, in a context of high demand of medical specialists like Latin America, it is unjustified to lose human resources because of gender bias.

### Limitations

One of the limitations of the current study is that it examines the “intention to choose” a specialty and not the “choice” itself. However, we believe there is no better method to measure the true choice preference, given that if the “choice” itself were studied, it could be affected by many factors not necessarily determined by a true preference for that specialty (for example, not achieving a specific score to apply for the specialty that they actually want to study causes students to choose another by “necessity” but not by preference). Similarly, it is possible that the question used to measure the outcome could have forced a choice; for this reason, we only considered those who named a specialty and not those who only mentioned wanting to choose a specialty but did not specify which. Third, it is necessary to state that we found significant differences according to gender between those excluded and included in the study. This fact could affect the magnitude of the association due to selection bias; however, the authors consider this issue to be an expected phenomenon, given that most of those excluded were in their first year and male. Additionally, not having included other socioeconomic, personal and specialty related variables as confounding variables can be questioned; however, these could not be studied because the primary survey was not conducted with this objective. Another limitation is the studied population is not representative of all of Latin America because it was not obtained probabilistically in each country. Proportions found in this study must be deemed with caution. However, given the large sample size and the fact that other data on this issue have not been collected from this geographic region, the authors consider that the benefits of these results outweigh the risks that the sample is not representative.

### Conclusions and Recommendations

There is a positive association between the female gender and the intention to choose Obstetrics/Gynecology, Pediatrics, Pediatric Surgery, Dermatology, and Oncology in Latin American medical students. Feminization is a growing phenomenon that is also present in Latin America. We recommend further studies that consider other factors that can influence the choice of medical specialty, such as academic experiences including rotations, exposure to a specific type of teachers, and the orientation of the university medical curricula. It is necessary to improve or change the factors that determine the choice of medical specialty according to gender, such that the same opportunities can be offered to men and women to develop any specialty.
